# Some Observations on Organic Alterations of the Heart, and Particularly on the Beneficial Employment of Iron in the Treatment of Such Cases

**Published:** 1846-07

**Authors:** 


Art. XI.—*Some Observations on Organic Alterations of the Heart, and particularly on the beneficial employment of Iron in the treatment of such cases.* By S. Scott Alison, m.d., m.r.p.l., and Physician to the Northern Dispensary.—*London*, 1845. 12mo, pp. 62.

This is a modest and sensible monograph. It is written with much moderation. There is no undue eulogy of the effects of iron in cases of heart disease: but the circumstances are carefully discriminated in which recourse to this agent promises benefit. There is much judiciousness in the doctrine, several times inculcated in these pages, that palliative treatment, even where curative is out of the question, is and ought to be a great object. The author likewise shows that some forms or modifications of organic change of the organ, are not so totally insusceptible of *cure* as has hitherto been by many supposed. We recommend this little volume as useful and opportune.

